# Review of "*Contact Mechanics and Friction: Physical Principles and Applications*" by Valentin L. Popov

**DOI:** 10.3762/bjnano.2.7

**Published:** 2011-01-25

**Authors:** Stanislav N Gorb

**Affiliations:** 1 Department of Functional Morphology and Biomechanics, Zoological Institute at the University of Kiel, Am Botanischen Garten 1–9, D-24098 Kiel, Germany

**Keywords:** adhesion, capillarity, contact mechanics, continuum mechanics, friction, lubrication, materials science, structural mechanics, system dynamics, tribology

Popov, V. L. *Contact Mechanics and Friction: Physical Principles and Applications,* 1st ed. Springer-Verlag: Berlin, Heidelberg, 2010. XV, 362 pages, ISBN 978-3-642-10802-0 (Print), 978-3-642-10803-7 (Online). doi:10.1007/978-3-642-10803-7

The book “*Contact Mechanics and Friction: Physical Principles and Applications*” is written by a theoretical physicist but from the point of view of an engineer. It covers an amazingly broad spectrum of topics ranging from atomic scale friction, continuum and structural mechanics, materials science, lubrication, adhesion, capillarity and system dynamics. It provides conceptual and computational tools for researchers in various branches of science which deal with the physics and mechanics of interfaces – from nanotechnology to earthquake research. One of the topics which seems particularly of interest to the author and which repeatedly finds its way into numerous examples and problems is the tribology of biological objects. Since Popov knows biological and biomimetic systems from his own collaborative research with biologists [1–3], the book is not only an excellent starting point for engineers and physicists working in biology but also for biologists studying friction and adhesion.

Properties of interfaces play a very important role in biology. In their evolution, organisms have evolved a variety of specialized surfaces. Some organisms live attached to a substrate; others can also move, fly, swim and dive. Many inter- and intracellular processes of biological cells use specific properties of interfaces. Understanding these is of major scientific interest, since it can give insight into the workings of nature in evolutionary processes. Beyond that, one can discover the detailed chemical and physical properties of materials, learn about their use as structural elements as well as their biological role and function. This knowledge is highly relevant for the technical applications of humans. Biologists have collected an immense amount of information about the structure of such living micromechanical systems and now find themselves in a situation in which a purely descriptive level of research is not always sufficient to extract new knowledge. This extremely complicated and interdisciplinary field of research can only be successful if it is considered though a prism of interdisciplinary theory. Such an interdisciplinary theoretical basis is provided by Popov’s book.

Because of an increasing scientific interest in understanding contact mechanics and friction/adhesion phenomena in biological systems, Popov’s book is very useful in experimental biology and biomechanics (for an example of a contact problem in biology see [4]). Biological and technical systems have many common features. First, the mechanical interaction occurs on identical length and force scales [5]. In both types of systems, surface properties – for example wettability, microstructure or surface chemistry – have a strong impact on the performance of the system [6]. Since biological examples are increasingly used to design novel technical systems (biomimetics), such as friction-induced worm-like motion, artificial joints for medical applications, gecko-inspired sticky tapes, etc., the book might aid in guiding such biologically inspired developments.

The book consists of 20 chapters dealing with, among others, the following problems: (1) non-adhesive contact problems, (2) adhesive contacts, (3) capillary forces, (4) contact between rough surfaces, (5) tangential contact problems, (6) Coulomb’s law of friction, (7) nanomachines: micro and nano-actuators, (8) frictionally induced vibrations, (9) thermal effects in contacts, (10) lubricated systems, (11) viscoelastic properties and friction of elastomers, (12) wear. The book is an excellent example of interdisciplinary science because it uses approaches from physics, engineering, tribology, materials science and some examples from biology. Because of its rigorous mathematical approach, it provides a first-class introduction to the principles of contact mechanics and tribology for specialists from different fields. However, it also contains many qualitative descriptions aimed at providing an overall understanding of the properties without any extensive mathematical treatment. This combination of qualitative understanding with numerous rigorously handled case studies may be of a special interest for biologists.

This book is clearly written, excellently illustrated, and therefore, can be used also by scientists specializing in biological surface science, biomechanics, experimental biology, and biomimetics. These scientists will find concise and precise models that aid quantitative description of surface phenomena in biology. The chapters of the book illustrate a few examples of contact problems in biology and give numerous examples of applications in contact mechanics to these kinds of problems (p. 25, p. 39, p. 48, etc.). Anyone who is doing research on biological contact problems or those who are particularly interested in the friction/adhesion phenomena in biology will find this book an excellent reference for its quantitative approach to these types of problems. Since the book provides worked solutions at the end of each individual chapter, it might serve as a very good extension to biomechanics courses.
